# Monthly Summary

**Published:** 1895-12

**Authors:** 


### Monthly Summary.

DISTURBANCE CAUSED BY DISEASES OF THE TEETH.—An interesting paper on this subject was read before the Harvard Odontological Society by Dr. Edgar F. Stevens, of Medford, Mass., in which a number of cases are cited to prove the important part that diseases of the dental organs play in nervous disturbances of different parts of the body. Few physicians, particularly those educated twenty or more years ago, think of the teeth as having anything to do with these pains, for the reason that in their student days they had scarcely any opportunity of learning anything about the teeth or oral cavity. "The time is coming," says Dr. Stevens, "when the student of medicine will have the same opportunities of studying dental pathology as he now has of studying diseases of the eye, ear, throat, etc." We should impress on our medical friends the fact that the intelligent dentists should be consulted by them in cases where disturbances of the nervous system do not readily yield to general treatment. —*Dominion Dental Journal.*

ABSCESSES.—I treated with a donaldson broach, Gates-Glidden drill, a great deal of warm, carbolized water, and a syringe, a long piece of cotton with carbolic acid on the end, and common sense; and I cured it, so far as I can see, and the patient is satisfied.

Now, as to how I filled it. I cleaned the roots out. It being a lower sixth year molar, I find the distal root has but one canal, the median or front root two, not two roots, but two canals. I take the oxid of zinc and mix with oil of cloves to a thin paste, or a little thicker, and after drying the roots as thoroughly as I can, keeping dry with napkin or dam, I place a little in the root and take a small piece of gutta-percha, warm it and force it in the root as far as I can, and then fill the roots with gutta-percha. I fill part of the crown with cement for a foundation and then build any kind of filling on that.

I have been fairly successful with dead or pulpless teeth, but not as much as I have where the pulp is a live one in a good state.

I have found as much decay in teeth this past year as I have in the years previous, with all due respect to those D. D. Ss. who would have us believe that they can prevent it, and I find that teeth decay around their fillings all the same.—A. A. Pearson in *Alabama Society*.

---

TO OPEN UP A BLIND ABSCESS.—With a small swab apply a drop of trichloracetic acid on the gum over the apex of the root; twist the swab gently, applying more acid till you reach the alveolus; with a spear-pointed drill go through the alveolus to the sac; with a swab apply carbolic acid (Calvert's No. 1, full strength), thoroughly permeating the tumor; if necrosis is present enlarge the opening and get direct contact with the diseased bone; syringe out the tract with pyrozone, and pump in concentrated lactic acid; saturate a small tampon of cotton with campho-phenique and aristol, and leave in the opening for two or three days till connective tissue begins to form.—A. C. Hart, in *Items of Interest*.

